# Prefrontal cortex development and its implications in mental illness

**DOI:** 10.1038/s41386-025-02154-8

**Published:** 2025-07-03

**Authors:** Caitlin M. Goodpaster, Chloe R. Christensen, Maryam-Batul Alturki, Laura A. DeNardo

**Affiliations:** 1 Physiology Department, David Geffen School of Medicine, University of California Los Angeles, Los Angeles, CA USA; 2 Neurosciences Interdepartmental Program, David Geffen School of Medicine, University of California Los Angeles, Los Angeles, CA USA; 3 Neurobiology Department, David Geffen School of Medicine, University of California Los Angeles, Los Angeles, CA USA

**Keywords:** Synaptic development, Stress and resilience

## Abstract

The medial prefrontal cortex (mPFC) plays an essential role in cognition and emotional regulation. The mPFC undergoes an extended development that is regulated by both genetic programs and activity-dependent processes. During this time, experiences feedback on developing mPFC circuits, allowing individuals to develop nuanced, age-appropriate responses to their environment. However, this protracted development also opens an extended window when adverse experiences such as neglect or maltreatment can alter the trajectory of mPFC development, leading to the emergence of mental health disorders like anxiety and depression. These disorders are characterized by excessive avoidance of perceived threats and impaired emotional regulation. These behavioral functions are encoded in the activity of mPFC neural circuits, particularly in mPFC connections with limbic centers like the basolateral amygdala and nucleus accumbens. To understand how mental health disorders emerge, it is critical to understand how frontolimbic circuits typically develop, and how early life adversity can alter their development. Here we review recent studies that examined the synaptic, cellular, and circuit development of frontolimbic circuits and the underlying molecular and activity-dependent mechanisms. We then review studies that measured the effects of early life stress on mPFC maturation and discuss the implications for therapeutic strategies.

## Introduction

The medial prefrontal cortex (mPFC) plays a key role in emotional regulation and cognitive processes, including decision making, working memory, and long-term memory [1, 2]. While mPFC is a highly heterogeneous structure that comprises functionally-distinct subregions, it is thought to have a unified role in integrating learned information about the environment with current goals in order to select appropriate behaviors [1]. The mPFC can influence many aspects of behavior through its diverse synaptic connections with hundreds of other brain regions. Through experience-dependent plasticity, mPFC circuits process incoming contextual information and appropriately bias activity in top-down pathways to influence behavior. In this way, mPFC promotes adaptive behaviors in a dynamic environment.

The mPFC undergoes an extended period of maturation that is likely critical to help animals transition through developmental milestones and establish nuanced responses to environmental stimuli [2, 3]. However, during this time, mPFC is vulnerable to environmental disruptions, which can interact with underlying genetic risk factors [4]. Early life adversity (ELA), such as maltreatment or neglect, is a major risk factor for developing neuropsychiatric disorders that are associated with dysfunctional mPFC circuits and related behaviors [5–8]. These disorders, including anxiety, depression, schizophrenia, and substance use disorder, arise early in life, often during adolescence [6, 9]. However, the mechanisms linking early adversity to later behavioral dysfunction are only beginning to be understood.

Here we review recent work on maturation of mPFC circuits, related behavioral changes, and the implications for understanding and treating mental health disorders. We discuss how developmental trajectories in mPFC and key interconnected regions influence behavioral changes across the lifespan, with a particular emphasis on changes occurring during adolescence. Throughout this review we will focus on three developmental stages, the juvenile period (rodent) /childhood (humans), adolescence and adulthood. We will define developmental stages following the American Academy of Pediatrics Association [10], categorizing infancy as ages 0–1, childhood as ages 2–10, adolescence as 11–21, and adulthood as 22 years and older. When referencing rodent studies, we will define infancy as postnatal day (P)0-21, the juvenile period as P21–27, adolescence as P28–45, and adulthood as P60 and beyond [2]. The onset of the juvenile stage coincides with increased independence from a caregiver, when rodents are typically weaned in the laboratory. In the literature, the boundary between the juvenile period and adolescence is not always consistent. Some groups consider adolescence to begin at weaning, others at the onset of puberty, and some at more broadly interpreted milestones, resulting in overlapping definitions of the juvenile and adolescent periods across groups. Even the onset of puberty, which is relatively easy to assess, can vary depending on sex and species. For instance, female mice can reach sexual maturity as early as P26, while male mice may not mature until around P40 [11]. In rats, females typically reach sexual maturity between P32 and P34, whereas males mature between P45 and P48 [12]. Additionally, studies on fear learning, reward, and social behaviors define adolescence as spanning from as early as P28 to as late as P60, though most research concentrates on the P28–P48 range [13, 14]. We have integrated this information in defining our selection of developmental stages.

Our review will focus on the frontolimbic system, including mPFC, the basolateral amygdala (BLA) and nucleus accumbens (NAc), which play key roles in emotional learning, reward seeking, and threat avoidance. Top-down mPFC pathways targeting BLA and NAc have different behavioral roles. Coordination of these pathways is key for selecting appropriate behavioral responses, including balancing approach and avoidance of environmental stimuli. Dysfunction in this system is strongly implicated in anxiety, mood, and substance use disorders, where approach and avoidance behaviors become maladaptive.

Emotional learning, reward seeking, and threat avoidance behaviors evolve across development in both humans and rodents, and frontolimbic circuits are highly conserved across species. Behavioral studies offer valuable insight into how the underlying circuits develop. Therefore, by aligning behavioral changes across species, and then examining underlying synaptic-, cellular- and circuit-level changes in rodent models, we can begin to understand the neurodevelopmental processes that drive normative behavioral changes, and how they may be disrupted by adverse rearing conditions that lead to human disorders. Past studies mostly examined frontolimbic circuit maturation and behavioral development independently. However, more recent work has integrated investigations of the two, now revealing circuit mechanisms that drive behavioral milestones. The work we discuss here builds a foundation of knowledge that can inform the development of targeted therapies to prevent or ameliorate circuit-level dysfunction associated with mental health disorders.

## Overview of mPFC postnatal development

Developmental changes in decision-making and cognitive control of both reward-motivated behavior and threat responding are associated with the maturation of the mPFC [15]. Aspects of cognition, including information processing speed, response suppression, and working memory, mature through late childhood and into adolescence [16]. These changes are accompanied by changes in the underlying mPFC activity. For instance, mPFC activity is more strongly distinguished between correct and error trials in a cognitive task in adults (18–27 years) compared to adolescents (13–17 years) and children (8–12 years) [17], consistent with popular models suggesting that the maturation of top-down control pathways influences behavioral changes across development [15]. Some studies have also observed peaks in cognitive performance in juveniles. For instance, juvenile (P26–27) rodents performed better than adults (P60–70) in a reversal learning task that depends on mPFC function [18]. This suggests that rather than driving a gradual improvement in cognitive flexibility, the slow mPFC development allows behaviors to be finely tuned to meet demands associated with different development stages [18].

### Synaptic development of the mPFC

In the human brain, mPFC cells and circuits undergo continuous changes from birth until early adulthood [2]. A massive wave of synaptogenesis in the PFC begins two months before birth and ends two months after [3]. mPFC synapse density peaks later than other cortical areas, around 3.5 years of age, and then declines until adulthood [19, 20]. In non-human primates, parvalbumin (PV)-positive cortical interneurons increase in number, and synaptic inhibition increases between childhood and adulthood (between birth and 4 years of age) [21, 22]. Myelination, which aids in rapid neurotransmission across long distances and also prevents axonal branching, begins during childhood (3–9 years) and increases into adulthood (>28 years) [23]. In line with these findings, adolescents (12–16 years) have more white matter and less gray matter in the prefrontal cortex compared to younger children (7–10 years) [24, 25]. These changes may be related to the timing of sensitive windows of development, when experience can have profound effects on brain structure and function, as described in the subsequent subsection [26].

**Fig. 1:**
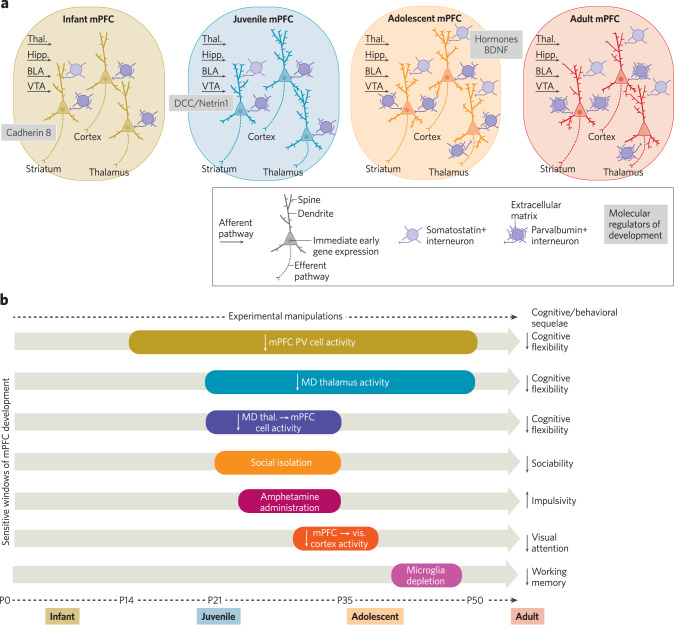
Molecular and activity-dependent mechanisms of mPFC circuit development. **a** Summary of synaptic and cellular changes in mPFC across four stages of development. **b** Summary of studies that manipulated mPFC circuit activity and examined adult sequelae.

Rodent studies enable detailed examination of cellular and synaptic changes during mPFC development. Through the infant to adolescent period (between P6–P30), mPFC pyramidal neurons elaborate their dendrites, receive and refine synaptic inputs, and establish long-range connections with distant brain regions [27]. During this time, the expression levels of immediate early genes decline as inhibitory interneurons mature and synaptic inhibition increases [27, 28]. An increase in dendritic spines early in adolescence (P26–30) coincides with increased ascending input from the hippocampus and BLA [27, 29]. While the intrinsic membrane properties of fast spiking interneurons abruptly mature in the second postnatal week, their microcircuits continue to develop into early adolescence (P30) [30]. Mirroring changes observed across postnatal human development, the density of perineuronal nets (PNNs) increases between adolescence (P35) and adulthood (P90) [31], mostly onto PV+ interneurons [32]. Likewise, these PNNs likely stabilize the synaptic architecture of the rodent mPFC [33, 34], as they are proposed to do in humans [35]. The mPFC is heavily innervated by neuromodulatory systems including by dopamine neurons in the ventral tegmental area (VTA) and substantia nigra, serotonin neurons in the raphe nuclei, and noradrenaline neurons in the locus coeruleus. As in humans, in rodents these systems mature at different rates and influence one another’s development [2] (Fig. 1a). See Klune et al. for a detailed review [2].

In contrast to sensory cortices, where the detailed mechanisms regulating circuit assembly have been the subject of decades of research, we are only beginning to understand the molecular mechanisms driving mPFC circuit development [36, 37]. Recent studies revealed that classical wiring molecules that play important roles in the spinal cord, sensory systems, and hippocampus are also important for wiring mPFC circuits. For instance, Cadherin-8 is critical for wiring prefrontal-striatal connections [38]. Deleted in colorectal cancer (DCC) and netrin-1 are critical for guiding axons from the VTA to the mPFC [39]. Brain-derived neurotrophic factor is important for the maturation of PV and somatostatin (SST)-expressing mPFC interneurons, and acts in a sex-dependent fashion [40]. Likewise, pubertal hormones regulate the maturation of synaptic inhibition in mPFC [41] (Fig. 1a).

### Sensitive windows of mPFC development

New studies have begun to reveal sensitive windows of mPFC development, when heightened plasticity allows interactions between experience and neurobiological factors to drive lasting changes in circuit function and behavior. These mechanisms promote adaptive functioning by enabling organisms to tune their brains to the demands of their environment. Interneurons have been widely recognized as key regulators of critical periods in sensory cortices. In regions like the visual cortex, critical periods are typically preceded by synaptic proliferation and characterized by a dramatic decrease in the excitation-inhibition balance driven by the maturation of inhibitory interneurons [42]. Several recent studies showed that the juvenile period through early adolescence is a sensitive window for aspects of mPFC development in mice. Chronically inhibiting activity in the mediodorsal (MD) thalamus throughout the juvenile period and adolescence (P20–50) caused a reduction in thalamo-prefrontal axon density and reduced excitatory drive onto mPFC neurons [43]. Affected mice had reductions in cognitive flexibility in adulthood that could be rescued by acutely increasing thalamic activity [43]. Inhibiting activity of mPFC-projecting neurons in the MD thalamus during a shorter period (P20–35) also impaired cognitive flexibility at P50 and caused cell-type-specific changes in prefrontal connectivity during adolescence (P35) [44]. The authors observed decreases in excitatory and inhibitory synapses onto prefronto-thalamic neurons, a selective increase in inhibitory synapses onto prefronto-cortical neurons, and layer-specific changes in synaptic connectivity onto mPFC-NAc neurons [44]. Inhibiting PV+ interneurons from P14–50 impaired mPFC circuit connectivity [45] and cognitive flexibility in adulthood (P90) [46]. Social isolation starting in the juvenile period and lasting into adolescence (P21–35) decreased the excitability of prefronto-thalamic neurons and increased inhibitory synaptic drive from SST+ interneurons at P90 [47]. Isolated mice had social deficits in adulthood (P63–69) that could be rescued by acutely increasing activity in prefronto-thalamic mPFC neurons [47]. Administration of amphetamines from the juvenile period to early adolescence (P22–31) disrupted DCC/netrin-1 signaling and led to ectopic growth of VTA-mPFC axons and altered cognition by adulthood (P75). These effects were only observed in male mice, highlighting a male-specific vulnerability to lasting changes in cognition following adolescent drug administration [48]. These findings shed light on why adolescent drug use may increase the propensity for addiction [49]. Thus, during early development, environmental changes that affect corticothalamic and interneuron activity may profoundly influence the trajectory of mPFC development (Fig. 1b).

Adolescence itself is also a sensitive window during mPFC development. mPFC neurons that project to the visual cortex have elevated activity during adolescence (P29–P37) [50] that is regulated by nicotinic cholinergic signaling [51]. Suppressing this activity specifically during adolescence (P29–P37) produced excessive loss of excitatory inputs to these neurons in adulthood (P65–P100) and impairments in visual attention behaviors [50]. Depleting mPFC microglia—non-neuronal cells that regulate synapse formation and pruning—specifically during adolescence (P42–47) led to cognitive sequelae (P84+) that were associated with changes in dendritic complexity and in the excitatory-inhibitory balance of mPFC pyramidal neurons [52]. Administering WIN, an agonist for the CB1 cannabinoid receptor, during early (P35–40) or mid- (P40–45) but not late (P50–55) adolescence impaired the maturation of mPFC GABAergic functions in adulthood (P65–95) and these changes could be rescued with acute administration of a positive allosteric modulator of GABA_A_ receptors [53]. Taken together, these studies revealed activity-dependent processes that regulate mPFC circuit development, as well as how they can interact with genetic mechanisms during specific developmental windows. Such findings are key for developing therapies, as many neuropsychiatric disorders arise due to interactions between genetic mutations and experience-dependent processes (Fig. 1b).

This body of work suggests that different mPFC circuit elements are sensitive in different developmental windows, providing important insights into how variations in the nature and timing of early experiences can lead to distinct outcomes with regard to cognition, behavior, and mental health. For example, prefronto-thalamic networks appear to be more sensitive during the juvenile and early adolescent periods, whereas prefronto-cortical connections and VTA inputs to the mPFC show greater sensitivity during adolescence. Inhibitory neurons and synaptic inhibition appear to be sensitive throughout both periods. Differences in sensitivities may be due to variations in the maturation rates of different cell types and their connections. These kinds of studies are key because they identify not only when mPFC circuit elements respond to changes in activity or pharmacological manipulations, but also how these changes impact behavior in adulthood. In doing so, this work reveals novel mechanisms through which drug taking, stress, or other adverse experiences may shape mPFC development, leading to later disruptions in cognition and emotional regulation. Some have proposed that puberty and the development of the dopamine system, which can promote interneuron maturation [54], can trigger these sensitive windows [26]. More research is needed to examine the complex relationships between pubertal hormones, dopamine signaling, and cell-type-specific development. Furthermore, given the complexity of mPFC connections, it will be critical to continue to define sensitive windows for different subcircuits.

## Exploration and reward circuit development

The mPFC sends extensive projections to the NAc and activity in this pathway has been linked to food reward seeking [55, 56], drug seeking [57], and context-dependent social learning [58]. The NAc also plays a critical role in shaping motivated behavior, serving to balance avoidance [59, 60], exploration [61], and risk-taking [62, 63] by integrating reward and threat-related signals to guide decision-making and adaptive responses. This complex interplay allows the NAc to modulate behavioral flexibility, reinforcing actions that maximize rewards while minimizing potential risks [64, 65]. Further, activity in the mPFC-NAc pathway encodes exploration in threatening environments [66], opposes threat avoidance behaviors [59, 67], and a subset of mPFC-NAc neurons can suppress reward seeking under threat of punishment [68]. Thus, this pathway encodes both threats and rewards and can dynamically regulate approach and avoidance behaviors in a context-specific manner. Projections from the mPFC to the NAc undergo protracted development throughout early life. How this pathway matures over time, how this influences behaviors that are crucial during adolescence, and how perturbations to these developmental trajectories can lead to behavioral disorders are only beginning to be understood. In this section, we review human and rodent studies investigating developmental changes in reward-seeking and exploratory behaviors and the underlying frontolimbic developmental mechanisms.

### Reward system development in humans

Magnetic resonance imaging (MRI) across development in humans has linked structural and functional changes in mPFC-NAc circuitry to changes in adolescent behavior. From early (9–12 years) to late (13–17) adolescence there is a slight increase in left NAc volume followed by an 8% decrease into early adulthood (18–23 years) [69]. When both hemispheres were included in the analysis, a steady decline in volume across adolescence and into adulthood (8–26 years) was reported [70]. During this time, gray matter volume rapidly declines across all subregions of the mPFC [71]. Reductions in volume across these regions represent rapid pruning and refinement of synapses that help establish adult-like networks [72]. Concurrently, during a probabilistic learning task, functional connectivity between the PFC and ventral striatum, which includes the NAc, strengthens from childhood (8–11 years) into young adulthood (18–22 years), and predicts developmental changes in decision making [73]. Adolescence (13–17 years) is also characterized by a drastic increase in NAc activation in response to reward [74], while frontal regions display diffuse activity patterns, more akin to that of children (7–11 years) than adults (23–29 years) [74]. These changes in reward processing within PFC and NAc coincide with peaks in reward sensitivity and motivation in adolescence (15-19 years) and young adulthood (18-23 years) [69]. Distinct reward processing, combined with a mature understanding of behavioral tasks and faster learning, gives adolescents a unique advantage when performing certain cognitive tasks [75].

There is evidence that exploration for informational gains is encoded similarly to reward in the brain, engaging both the ventral striatum, including the NAc, and the ventromedial PFC [76–78]. Exploration facilitates the acquisition of new information, allowing individuals to learn the rules of their environment. This expanded mental framework increases the range of available choices that can be utilized when making future decisions. Recent work has shown that explore-exploit strategies change across development, with children and adolescents preferring exploration strategies compared to adults [79–82]. In a multi-armed bandit task where participants chose amongst a fixed number of options, each of which produced a fixed reward, adults (21–72 years) briefly explored the options before consistently choosing the one that produced the highest reward. Children (4–12 years), on the other hand, were more likely to continue exploring all options, which proved beneficial when reward contingencies were switched [79, 80]. In a value-based decision-making task, adults (18–27 years) exhibited a more exploitative decision strategy than children (8-12 years) which was associated with a stronger aversion to uncertainty in adults (18–27 years) [83]. As children know less about the world than adults, it may be advantageous to explore more during childhood to enhance learning, and then gradually shift to a more exploitative decision strategy as individuals gain knowledge through experience [83].

During adolescence, increased exploration is also associated with a propensity to engage in risk-taking behavior [2, 67, 84–88]. Risk-taking behaviors involve potential exposure to harm, loss, or failure in pursuit of a perceived reward. They reflect the ability to tolerate ambiguity, make decisions, and integrate that information into a working model of the world. Typically, risk-taking during adolescence is evolutionarily advantageous, driving behaviors and decision making that expand knowledge, skills, and social networks essential for long-term survival and reproductive success. While risk-taking is an important part of learning the rules of new environments, excessive risk-taking can have dire consequences. In this age group, differences in sensation seeking and impulsivity are thought to lead to higher rates of dangerous activities, including binge drinking, illicit drug use, rebellious behavior, and driving while under the influence, resulting in a 200% increase in mortality rates in adolescence [89]. The ability to incorporate risk and reward into risky decision making in gambling tasks matures by late childhood (8–9 years) [90]. However, children (8–10 years) and adolescents (12–14 years) are more likely to choose risky options in ambiguous gambles [91]. In a virtual driving game [92], adolescents (14–18 years) displayed heightened risky decision making, and peer presence enhanced activity in reward-related brain regions, including the ventral striatum [93], in a manner that correlated with risky driving choices. Higher levels of adolescent (12-29 years) rebellious behavior was correlated with slower decline in mPFC volume, a proxy for slower maturation of this region [71].

**Fig. 2:**
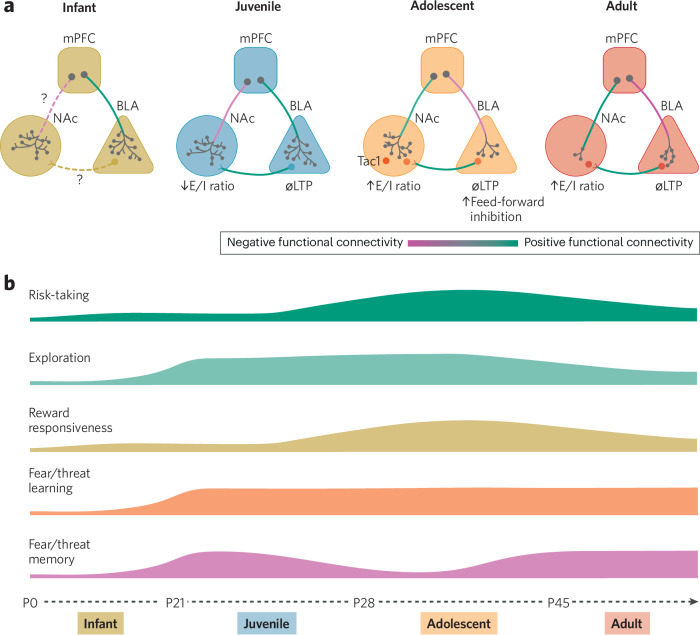
Structural and physiological changes in frontolimbic circuits and behavior across development. **a** Depiction of the developmental trajectory of mPFC-NAc and mPFC-BLA pathways from infancy through adulthood. Transition from positive to negative connectivity in functional (f)MRI studies depicted in red to green. Synaptic innervation and axon terminal density visualized with gray branching and dots in each target region. Dotted lines represent unstudied circuits. **b** Concurrent fluctuations in risk-taking behavior, exploration, reward responsiveness, fear/threat learning and fear/threat memory across all stages of development.

Together, these findings suggest that adolescence is characterized by a combination of heightened reward sensitivity and still-developing prefrontal modulation of the NAc. This imbalance promotes reward-seeking, increasing tolerance for uncertainty in situations that involve a potential reward. This plays a large role in promoting both exploration and risky decision-making during this unique stage of development. While human studies offer valuable insight into broad structural and functional changes in these regions, they lack the resolution needed to map circuit-specific synaptic modifications or uncover their mechanistic role in reward-seeking behaviors. In contrast, animal models allow us to apply advanced techniques to dissect synaptic changes across development at the circuit level, to determine causal roles of specific cell types, and to deepen our understanding of how these changes promote adaptive behavioral strategies in adolescence (Fig. 2a, b).

### mPFC-NAc synaptic development

While human studies have provided some insight into how mPFC and NAc volume and connectivity change across development, studies using rodent models—paired with advanced viral tools—allow targeted study of synaptic development in the mPFC-NAc pathway. This research provides a deeper understanding of the synaptic and physiological processes driving developmental trends observed in humans. Using a viral tracing strategy that labels axons and putative presynaptic puncta in mice, Klune et al., showed that mPFC-NAc axonal projections decrease in density from the juvenile (P23) period through adolescence (P35) and into adulthood (P60) [67]. In rodents between P4 and P30, excitatory postsynaptic potentials in NAc medium spiny neurons steadily increase in frequency, while inhibitory postsynaptic potentials increase in frequency between P4 and P12, and then remain steady [87]. A similar pattern emerges in the mPFC-NAc pathway. Stimulation of mPFC terminals in the NAc leads to increasing EPSC amplitude, no differences in IPSC amplitude and a significant increase in excitatory to inhibitory ratio from the juvenile (23) to adolescent (P35) period and into adulthood (P60) [67]. Reductions in mPFC and NAc volume, decreased mPFC axons in the NAc, and strengthening of synapses in this pathway could help explain similar changes observed in human studies. These findings indicate that physical pruning combined with synaptic strengthening in the mPFC-NAc pathway may influence behavioral transitions in exploration and risk-taking from early childhood to early adulthood (Fig. 2a).

Complementary to human data, risk-taking behavior also peaks in adolescent rodents. When given a choice between a lever with a small reward and one with a larger reward that carries an increasing risk of shock, adolescent (P35-51) rats choose the larger reward more frequently than adults (P77+), and they tend to interact more with the reward itself rather than the cue that predicts it [86]. Increased interaction with the cue, as opposed to reward, is associated with increased risk-taking in adult (~P72) rats, however, in adolescence (~P30), risk-taking is greater despite reduced interaction with the cue [94]. This suggests that the neural mechanisms driving risk-taking during adolescence are unique from those engaged in adulthood. When rats are trained to withhold a response to receive a reward, adolescents (P37–46) make more impulsive, premature actions than adults (60–71) [95]. Impulsivity can contribute to risk-taking behavior and is driven by increased activity in orbitofrontal cortex projections to the dorsomedial striatum and greater functional connectivity between the two regions in adolescents (P44–46), but not adults (69–71) [95]. Conversely, adolescents (P30–40) have immature connections between the medial orbitofrontal cortex and NAc that result in a reduced risk assessment behaviors in the elevated plus maze, a measure of anxiety-like behavior in mice [96]. mPFC-NAc has been shown to play a role in motor impulsivity, rather than risk-taking, in adults [97]. However, further investigation is needed to parse out the role of this circuit in the adolescent brain. These studies highlight the complexity of the neural underpinnings of risk-taking behaviors during adolescence. Different subregions of the PFC send projections to different parts of the striatum, each of which matures at different rates, differs in activation in adolescence, and plays a role in different behavioral aspects of risky decision making. By mapping the maturation of these different pathways and identifying sensitive periods of plasticity we will be able to better develop targeted interventions and preventative strategies aimed at fostering adaptive risk-taking, and reducing harmful behaviors in at-risk populations (Fig. 2b).

Behavioral studies of exploration and novelty in rodents have yielded mixed results. While adolescent (P38) rodents display shorter latencies to explore a novel object compared to adults (P70) [98], they spend the same time exploring novel odors [67]. On the other hand, juvenile (P23) mice exhibited more head dips in a hole board assay compared to both adolescents (P35) and adults (P60) [67]. Thus, while adolescent rodents, like humans, exhibit elevated risk taking, the relationships between exploration, novelty seeking, and risk-taking remain unclear.

Adaptive decision-making depends on a balance between approach behaviors—such as exploration and risk-taking, that drive goal achievement and positive outcomes, and avoidance behaviors that safeguard against potential harm. In adolescence (P35), avoidance is reduced to help promote exploratory behavior and is driven by higher activity in the dorsomedial PFC (dmPFC)-NAc pathway compared to adults (P60). Increased activity in this pathway also drives increased activation of downstream Tachykinin Precursor 1-expressing neurons in the NAc, which are known to promote approach behaviors towards aversive stimuli [99]. Taken together, these data suggest that heightened activity in the dmPFC-NAc pathway reduces avoidance behavior in adolescents, biasing behavior towards exploratory actions that are valuable in learning about the world and establishing independence at this age.

## Fear learning and threat circuit development

Fearful behaviors oppose risk-taking and are developmentally regulated. While avoiding threats can temporarily restrict opportunities to gain rewards, appropriate levels of threat avoidance minimize exposure to potential harm, promoting survival so an individual can seek rewards in the future. The BLA is involved in emotional processing, including encoding cue-threat associations and regulating emotional responses to threatening cues. While the BLA plays a key role in both reward and fear learning in adults, most developmental studies to date have focused on its role in fear learning and threat-induced behaviors, so we focus on those. When an individual perceives a potential threat, the BLA becomes active. It then engages downstream nuclei through its projections to initiate an appropriate behavioral response. For instance, BLA projections to the central amygdala are important for the generation of freezing behavior [100], whereas BLA projections to the NAc are important for active avoidance behaviors [101]. Input from the mPFC shapes BLA activity, influencing the maintenance and expression of threat responses. The mPFC-BLA pathway undergoes prolonged development, characterized by dynamic changes in its structure, physiology, and functional roles, which may evolve to support behavior strategies that are vital at each age. In this section, we review developmental changes in threat-induced behaviors in humans and rodents. We summarize recent advancements in human fMRI studies that investigated the neural correlates of these behaviors. We also highlight rodent studies that investigated mPFC-BLA synaptic and cellular maturation and performed rigorous circuit manipulations to link activity causally to behavior across development, adding nuance to current models of the neurodevelopmental mechanisms driving transitions in threat-induced behaviors.

### Maturation of threat responding in humans and rodents

While fear learning develops at a young age, the behavioral expression of fear memories is developmentally regulated. Many studies use Pavlovian conditioning to study the behavioral expression of fear. In cued fear conditioning, a predictive cue (e.g., a tone or an odor) is repeatedly paired with an innately aversive stimulus so the cue itself comes to elicit a fearful response. In contextual fear conditioning, a context is paired with an aversive stimulus, so individuals learn that a context or environment is threatening. Fear conditioning is possible in both rodents and humans, allowing researchers to closely compare the neural mechanisms. Other studies also look at innate threat responses in rodents using assays like the open field or elevated plus maze. In these assays, rodents tend to avoid the open parts of the arena where they are more vulnerable to predation.

Threat avoidance behaviors change across development and rely on conserved brain mechanisms in humans and rodents [67, 102]. In humans (3–5 years) and rodents (P8), exposure to a shock-conditioned odor can elicit paradoxical approach behaviors, but only if they were conditioned in the presence of a parent [103, 104]. In rats, this phenomenon continues until weaning (P21), which is analogous to late childhood in humans [103]. This developmental shift in behavior aligns with the development of the BLA and allows individuals to explore and distinguish between safe and dangerous aspects of their environment, all within the secure context provided by a caregiver [103]. Later in development, juvenile (P23), adolescent (P35), and adult (P60) mice can all learn to avoid a cued foot shock, but after learning took place, juveniles and adolescents displayed significantly less cued threat avoidance than adults [67]. In an elevated plus maze, juvenile (P35) and adult (P61) mice showed a strong preference for the closed arms, whereas adolescents (P48) explored the open and closed arms equally [105]. Another study did not observe differences in time spent in the open arms, but found that in early adolescence (P30–40), rats showed fewer cautious risk assessment behaviors before entering the open arms compared to adults (P70–90) [96].

The ability to form and express fear memories is key for adaptive threat avoidance and also changes across development. Studies in both rodents and humans have shown that the ability to form fear memories emerges during infancy (~P13-17) [106–108]. In rodents, aversive cue-conditioned responses are largely suppressed before P10 [109]. After P10, aversive-conditioned responses emerge but are regulated by the presence of a caregiver until ~P15 [103]. Contextual fear emerges slightly later, between P15-P17 [108]. However, episodic memories formed in infancy and early childhood are rapidly forgotten through processes known as infant- or childhood amnesia [108]. The ability to form lasting fear memories emerges during the juvenile period (P23), although memories formed during this period are less likely to undergo spontaneous recovery following extinction, suggesting they too are more fragile than memories formed later in life [110]. Interestingly, although the ability to form and recall long-term memories is already established, some studies found that adolescents (P^29^–^39^) display a temporary reduction in fear memory expression [67, 111]. Of note, others did not [107, 108]. These discrepancies may be due to differences in fear conditioning protocols or assessment of freezing. It could also be the case that when the threat is more ambiguous [67] (milder shock with the ability to avoid), adolescents may favor increased risk-taking and exploration at the expense of fear-based strategies.

Some of these behavioral changes likely reflect differences in relationships with caregivers [112]. While infants and juveniles rely on caregivers for protection, adolescents are establishing independence. Memory is an important tool for future decisions, but forgetting fearful experiences from very early in life may prevent the entrenchment of negative behaviors that are no longer useful or appropriate later [113]. Indeed, in section four, we will discuss the “stress acceleration hypothesis”, which describes how early chronic stress can lead to early closure of the window for childhood amnesia, which may put individuals at risk for developing fear and anxiety disorders later in life. Reductions in the behavioral expression of learned fear and threat avoidance in adolescence may enable exploration and trial-and-error learning so individuals can adapt to unfamiliar situations [114]. Many of these changes in behavior align with the developmental timeline of the mPFC and the frontolimbic system overall, suggesting these neural circuits play a causal role [2, 115]. In the following section, we review recent studies of the neurodevelopmental mechanisms that may drive behavioral changes in approach and avoidance behaviors (Fig. 2b).

### mPFC-BLA synaptic development

A shift in the encoding of fearful stimuli has been observed in human development. In children (4–9 years), the amygdala exhibits strong reactivity to emotional stimuli (i.e., fearful faces) before mature connections with the PFC are established [116]. In childhood (7–12 years), a positive correlation between the PFC and amygdala emerges in response to fear. By age 10, PFC-amygdala connectivity switches to being negative, and becomes progressively more negative into young adulthood (19–25 years) [116, 117]. This change in connectivity is paralleled by a decrease in amygdala reactivity [116], and some suggest it reflects the increasing inhibitory role of the PFC over the amygdala [118]. This is supported by animal work that describes weak recruitment of inhibition in the lateral amygdala and weak excitatory PFC-driven activity in the basal amygdala in adolescents (P39) compared to adults (P72–75) [119]. Less regulation of BLA, characterized by weaker prefrontal excitatory inputs, absence of synaptic plasticity, and changes in inhibitory recruitment, all may serve an adaptive function. Unique organization of this circuit may allow for greater flexibility in threat assessment and reduce the persistence of fear memories during a period of heightened exploration and risk-taking.

In rodents, synaptic maturation of the mPFC-BLA pathway continues until early adulthood. In mice, mPFC axons arrive in the BLA by P15, later than in other targets, including the claustrum, thalamus, and striatum [120, 121]. The appearance of mPFC axons within the BLA coincides with a large increase in the frequency of spontaneous excitatory and inhibitory synaptic events in the BLA [120]. These changes align with weaning, when the mice are separated from their mother, and may need to engage fear learning circuits to promote survival. While the number of mPFC-BLA projection neurons remains stable through adolescence (P35) [122], the density of their axons in the BLA varies based on age and on the specific subregion of the mPFC that was examined. For instance, a study that targeted the projections of the entire mPFC reported modest increases in BLA innervation through late adolescence (P15–45), followed by a slight decline in adulthood (P60) [120]. However, axons originating from the ventral mPFC stably innervated the BLA through adolescence (P25–45) and then their density declined in adulthood (P90) [121]. Axonal projections from the dorsal mPFC to the BLA were reduced in density between juvenile (P23) and adolescence (P35) stages, and then plateaued between adolescence and adulthood (P60) [67]. mPFC-BLA excitatory synapses strengthen between adolescence (P39) and adult (P72-75) stages [119]. Feed forward inhibition in the mPFC-BLA pathway is also relatively steady between infant (P15–21) and adult (P60) ages, except for a transient surge in early adolescence (P30) [120]. Changes in the balance between synaptic excitation and inhibition in adolescents may contribute to their reductions in fear and avoidance behaviors.

Both auditory fear conditioning and threat avoidance learning induce synaptic plasticity in the mPFC-BLA pathway in adults, resulting in enhanced excitatory signaling, fear memory encoding, and robust fear memory recall [123, 124]. Juveniles (P23) and adolescents (P35) lack synaptic plasticity in this pathway, despite similar levels of behavioral learning in a threat avoidance assay [67]. The same study also found that after animals learn to avoid a cued threat, activation of mPFC in adults preferentially recruits a serial BLA-NAc circuit known to be key for threat avoidance, but this preferential activation was absent in juveniles (P23) and adolescents (P35) [67]. Early-adolescent (P29) mice also lack mPFC-BLA synaptic plasticity following contextual fear conditioning [111]. Adolescents (P28–40), as opposed to adults (P71–81), also have a marked reduction in local field potential (LFP) summation in the BLA following fear conditioning [125]. While PFC stimulation dampens excitatory inputs from the thalamus following fear conditioning in adults (P71–81), this regulatory mechanism is absent in younger animals (P28–40) [125]. Interestingly, while contextual fear conditioning does not induce synaptic potentiation within the BLA during adolescence (P29), this plasticity emerges two weeks following learning [111]. This delayed emergence of plasticity suggests that while fear learning occurs behaviorally, the underlying neural consolidation processes may be temporally shifted compared to adulthood (Fig. 2a, b).

## Early life adversity: effects on circuits and behavior

ELA is a major risk factor for the development of anxiety and depression, conditions that often manifest during adolescence and are characterized by enhanced avoidance behavior. ELA can include neglect, maltreatment, socioeconomic hardship, exposure to violence, or other forms of chronic stress. We focus on studies of altered caregiver relationships, as parental presence can enhance or reduce children’s response to stress [126–129]. By altering activity in the hypothalamic-pituitary-adrenal axis in sensitive windows of development, ELA is thought to shift the developmental timing and function of regions that express high levels of glucocorticoid receptors, especially in the frontolimbic system [130]. For instance, parental presence can attenuate both mPFC and central amygdala reactivity to an aversive stimulus [129, 131]. Thus, parents can modulate activity in emotion hubs in the brains of offspring, but as we discuss in the following section, these effects are age-dependent [129]. Here we review studies that examined the impact of altered parental behavior, most notably neglect or maltreatment, on mental health. We review parallel studies of human and rodent frontolimbic circuit development as it relates to behavioral changes, and discuss how they inform avenues for preventing and treating stress-related disorders in at-risk populations.

### Behavioral and neurocognitive changes following ELA

**Fig. 3:**
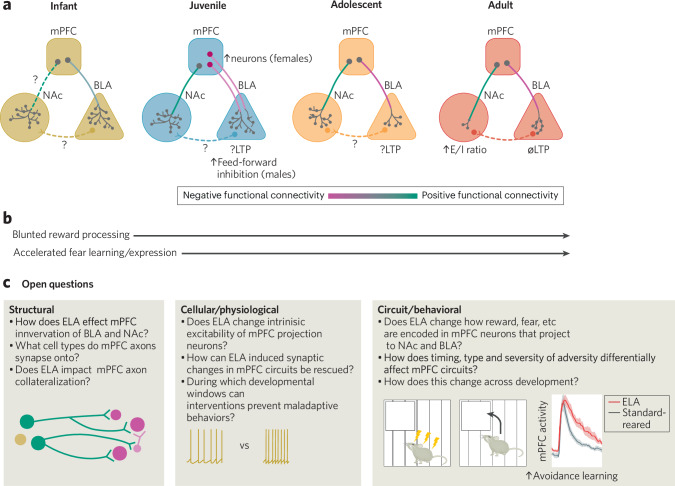
Changes in frontolimbic circuitry as a result of ELA and future research directions. **a** Differences in the developmental trajectory of mPFC-NAc and mPFC-BLA circuits as a consequence of ELA. We use the same gradient coloring scheme to illustrate connectivity measured in fMRI studies and note female-specific changes in pink, and male in blue. Dotted lines represent circuits that have not been studied in the context of ELA. **b** Blunted reward responsiveness and accelerated fear/threat behaviors as a consequence of ELA are seen throughout development. **c** Proposed future directions for research to further understand how ELA alters the developmental trajectory of frontolimbic circuits and identify sensitive windows for therapeutic intervention.

There is robust evidence supporting that ELA blunts reward processing beginning in childhood and persisting into adulthood. This dysregulation can result in an increased risk of anhedonia, impulsivity, or maladaptive reward-seeking behaviors. Over time, these changes contribute to a higher susceptibility to mood disorders, addiction, and other mental health challenges. Changes in reward processing as an effect of ELA have been extensively reviewed elsewhere [6, 132], but we will briefly outline the main findings. Overall, data suggest that children who experience adversity in the form of abuse [133] or institutionalization [134, 135] exhibit reduced reward responsiveness, slower reward learning and have difficulty updating value when reward contingencies are switched. Reward responsiveness in humans can be measured using a probabilistic reward task in which they are presented with one choice that leads to reward most of the time and another that rarely results in a reward [136]. Over time, participants learn to bias their response to the choice that results in the higher reward [136]. Individuals with anhedonia consistently display reduced bias that can predict neuropsychiatric symptoms [137–139]. In a modified version of this task, rats that experienced unpredictable maternal care reduced their responses towards the high reward option and didn’t display a preference for a sucrose reward [140]. A similar result was seen following maternal separation in mice [141]. These studies are unique in that they use a translational conserved assay to study reward responsiveness and find consistently blunted reward processing in clinical populations and animal models of ELA. Continued alignment of behavioral assays between humans and rodents will enhance translational relevance and harness mechanistic insights from rodent research to develop effective therapeutic interventions for humans (Fig. 3a, b).

Blunted reward processing plays a significant role in shaping risk-taking behavior among individuals who experience ELA. Research indicates that heightened risk-taking tendencies depend on whether a decision involves a potential gain or loss. Notably, maltreated children exhibit altered decision-making only when faced with potential losses [134, 142]. Specifically, in a gambling task they (9–11 years) show insensitivity to increasing loss magnitude, a factor that typically deters most children from a given choice [134]. Another study using a similar task found that maltreated individuals (18–25 years) show low levels of risk adjustment, indicating their decisions are not as sensitive to risk levels during a trial [143]. Unlike their peers, maltreated individuals struggle to adjust their behavior to minimize negative outcomes. These findings suggest that adverse experiences disrupt an individual’s ability to accurately assess the value of negative outcomes and adjust their behavior accordingly. Impaired risk assessment likely plays a role in the increased tendency to engage in harmful behaviors, such as drug use [144], excessive alcohol consumption [49, 145], and risky sexual activity [146], all of which are highly prevalent among individuals who have experienced ELA. Thankfully, interventions have the potential to reduce deficits. In a group of maltreated adolescent females (15–17 years), participation in a skills-based, family-oriented program designed to reduce risk-taking behaviors, such as delinquency and substance use, in middle school helped rescue deficits in decision-making [142].

Children and adolescents who were institutionalized in early life also tend to have elevated anxiety, which is characterized by an intolerance for uncertainty [147]. Those with high intolerance for uncertainty often struggle with ambiguous, unpredictable, or unknown situations and respond with high levels of avoidance. Adolescents (P14–20) who experienced maltreatment have been found to have higher levels of self-reported stress during uncertain situations, and this may mediate an observed decrease in exploratory behavior [148]. In older adolescents (18–19 years) presented with either threatening or ambiguous social images in an fMRI scanner, neural representations of those stimuli in the mPFC, BLA, and NAc were more similar in individuals who experienced ELA [149]. Thus, in individuals with a history of ELA, ambiguous stimuli activate threat response circuits, likely triggering avoidance responses in circumstances when typically raised individuals might perceive safety and choose to explore. Excessive threat avoidance during adolescence may be especially problematic. This is a crucial period for exploration, which plays a vital role in learning the nuanced rules of one’s environment, enabling adaptive decision-making in the future.

Increases in avoidance behaviors during adolescence do not happen in a vacuum and provide a window into how the developmental *trajectory* of mPFC circuits is altered following ELA. In 2016, Callaghan and Tottenham posed a “stress acceleration hypothesis” [150]. They argue that adverse experiences early in life drive aspects of the brain’s emotion circuitry to develop at faster rates, leading to premature adoption of avoidance strategies in development. Several lines of research in rodent literature support this hypothesis. As mentioned earlier in this paper, typically reared rats will approach an aversive stimulus in infancy in the presence of their mother, and then switch to avoidance after P10, following synaptic maturation in the amygdala [103]. In rats reared with limited bedding and nesting (LBN), a model of resource scarcity that induces ELA by altering maternal caregiving, this switch occurs earlier [151], suggesting that this experience accelerated the development of brain systems mediating responses to learned aversive stimuli. Infant mice (P16–17) who were separated from their mother often also show high levels of freezing 7 and 14 days after fear conditioning, a time when typically reared mice would exhibit infantile amnesia [152]. LBN conditions also led to a reduction in fear expression at P21. Although this study lacks an adolescent time point for comparison, these results suggest that temporary suppression of fear expression, typically observed in adolescence (P29-P39) [111], occurs earlier in those who experience adversity. Observation of rapid maturation of pathways seems specific to frontolimbic emotion circuits, as similar patterns are not observed in other brain areas [153]. This phenomenon promotes behaviors that prioritize survival at the expense of exploration. Adolescents who grow up in unpredictable environments lack the stability and security needed for healthy exploration and learning, ultimately curtailing their ability to develop adaptive decision-making strategies for navigating a complex world later in life.

While ELA markedly increases fear and avoidance during the adolescent period, adults paradoxically exhibit a reduction in these behaviors. Several rodent studies have found that LBN significantly reduces the ability of adults (>P70) to recall and express context fear memories [154–156]. This memory deficit is not due to differences in learning, as freezing levels during the training session are equivalent. Glucocorticoid receptor antagonists, administered during the adolescent (P28–30) period, can rescue memory deficits [156, 157]. In auditory fear conditioning, however, the results are not as clear, with some studies showing decreases in freezing in only males [154], both sexes [156], or in neither [157]. Interestingly, during recall, adult (>P120) male mice who experience LBN show elevated levels of freezing in between the threatening tones, but less freezing during the tone compared to controls [157]. In another study, adult (>P70) mice who experience LBN show increased startle responses to white noise, an ambiguous cue, as well as to a cue paired with an aversive stimulus [158]. Together, along with the human data [149], suggests that ELA reduces the ability for this group to modulate their response based on threat imminence, and instead express a moderate level of generalized fear (Fig. 3a, b).

The discrepancies between context and auditory fear conditioning may be due to the different brain regions involved. The hippocampus is required for encoding and recalling contextual memories. In this region, ELA reduces synaptic plasticity, dendritic arborization and dendritic spine density (see Baram & Birnie, 2024 for more comprehensive review [8]). This could indeed explain the memory deficits present in behaviors that rely solely on contextual information, however, auditory fear conditioning is more heavily dependent on BLA circuits, which do not show the same structural changes. Additionally, the type of adversity experienced (i.e., maternal separation versus resource scarcity), as well as differences in shock intensity and protocols for fear conditioning paradigms, can impact the behavioral changes. Standardization of protocols and replication efforts across labs would help us gain insight into how different modes of adversity can impact neural circuitry and the behaviors they control.

### ELA: impacts on frontolimbic neurodevelopment

In humans, changes in functional and structural connectivity of mPFC-BLA circuits may underlie changes in emotional processing in those who experience ELA. When viewing emotional images, fMRI studies have revealed that children (6–10 years) usually display a positive relationship between mPFC and amygdala, and that this switches to negative in adolescence (10–17 years) [159]. However, this switch occurs early in children (6–10 years) who have been previously institutionalized [159], maltreated [160], or experience post traumatic stress disorder [161]. In an MRI task where children and adolescents (7-16 years) were conditioned to associate a particular image with an aversive sound, those who experienced ELA had a negative correlation between multiple PFC subregions and the amygdala when viewing the aversive image, when compared to controls [162]. In previously institutionalized children, this connectivity pattern was mediated by the hormone cortisol, suggesting that stress-induced changes shape development of this circuit [159]. Those that were institutionalized also displayed increased anxiety overall, although this was mitigated by greater negative connectivity between mPFC and amygdala. Along the same lines, the transition to mPFC-amygdala negative resting state functional connectivity was associated with reductions in anxiety among socioeconomically disadvantaged youth (5–25) [163]. This suggests that accelerated maturity in this circuit partially ameliorates anxiety phenotypes, and that interventions that enhance this negative connectivity may further improve mental health outcomes in individuals who experienced early life stress.

Similar changes are also observed in resting-state fMRI in both infants and children who experienced ELA. Infants (5 weeks old) whose mothers experienced gestational stress during the last two trimesters of pregnancy display less positive functional connectivity between amygdala and PFC [164], a pattern that typically does not arise until later in development. Decreased functional connectivity was also associated with increased structural connectivity between these two areas [164]. Another study found that 4–7-year-old children who had had more stressful experiences had weaker positive coupling between the PFC and amygdala during resting state [165]. Interestingly, weaker connectivity was also associated with aggressive behavior and attention problems in these individuals [165]. Together, these findings suggest that while the PFC and amygdala typically exhibit positive connectivity during early life, exposure to stress in early development disrupts this relationship. The severity of stress appears to influence these changes, with mild stress weakening connectivity, while extreme stress—such as early institutionalization—accelerates the development of regulatory control over emotions, potentially as an adaptive mechanism to dampen excessive neural responses to future threats.

Although extremely valuable, individual differences among human participants, challenges with recruitment and retention for longitudinal data collection, and ethical concerns about studying threat-induced behaviors in young children limit our understanding of how ELA affects the brain. Animal models allow manipulation of neural activity during behavior, enabling precise dissection of circuit function to uncover the cellular and molecular processes driving ELA-induced behavioral changes. Rodent studies investigating ELA-induced brain and behavior changes have focused largely on adult outcomes, but there have been recent advances in our understanding of how mPFC-BLA synaptic changes across development as an effect of stress.

In agreement with the human literature, mice that experienced LBN display weakened mPFC-BLA resting state connectivity at P18 [166]. Reduced connectivity persists into adulthood (P74-75) and is associated with reduced fear extinction. LBN also leads to transient low-theta oscillatory hyper-coupling between the mPFC and BLA in infant (P18–20), but not adolescent (P43–47), male rats [167]. In this group, mPFC spiking and local theta entrainment of spike firing was also disrupted. These differences were not seen in females, yet both sexes had increases in activity of non-GABAergic BLA cells when compared to controls. In another model of ELA, maternal separation, connectivity was reduced between the infralimbic, but not prelimbic, subregion of the mPFC and the BLA in late adolescence (P38–48), but only in females [168]. Maternal separation has also been shown to disrupt prefrontal-amygdala network synchronization by increasing prefrontal feed-forward inhibition on BLA in young male rats (P14–21) [169]. These findings emphasize how early-life care can significantly influence the development of prefronto-amygdalar circuits, potentially in a sex-dependent manner. Even transient adversity during very early life can alter mPFC-BLA communication, with effects emerging as early as the juvenile and adolescent stages. This underscores the existence of a sensitive window during which targeted interventions could help redirect the trajectory of emotion circuit development toward a more typical course. However, the type of stressor employed, as well as differences in protocol and implementation across labs cause discrepancies in specific results. Establishing standardized protocols and prioritizing replication efforts would enhance the consistency of findings, allowing for a more robust and comprehensive understanding of how different modes of adversity lead to aberrant behavior (Fig. 3a).

Compared to mPFC-BLA circuits, our knowledge of how ELA affects the development of mPFC-NAc is limited. The few studies that have investigated this indicate that ELA blunts activation of reward circuitry. Previous institutionalization for more than 6 months reduces activation of the ventral striatum when anticipating a reward in the Monetary Incentive Delay Task [170]. In controls, ventral striatum activity increases proportionally with anticipated reward magnitude, suggesting that blunted activity observed in the ELA group reflects a reduction in sensitivity to reward value [170]. Resting state mPFC-NAc connectivity is overall negative in this group of control children (6–10 years) and adolescents (11-18 years), but is positive in previously institutionalized youth [171, 172]. Positive resting state functional connectivity is also associated with more social problems, especially during adolescence [171]. These studies provide insight into how extreme forms of ELA can alter corticostriatal circuits, however, many children experience less severe forms of stress, which can also influence brain development. Indeed, there is a negative correlation between the number of (less severe) stressors experienced and NAc activation in response to reward anticipation in 10-years-old [173]. At this age, activity in the mPFC and anterior cingulate cortex predicted stress reactivity to an acute stressor three years later [173]. Severity of adversity is also negatively correlated with integrity of accumbofrontal white matter tracts in adolescence (12–17 years) [174]. However, there are some biomarkers that protect against negative behavioral outcomes. Activity of NAc and amygdala during reward anticipation buffers the association between life stressors and depressive symptoms in adolescents (13–19 years) [175]. And greater orbitofrontal cortex thickness and corticolimbic white matter integrity protects against ELA-induced anxiety (18–22 years) [176]. During adolescence, increased activation of reward circuitry, including the mPFC and NAc, perhaps combined with decreasing amygdala reactivity, typically fosters adaptive behaviors that encourage exploration and information gathering about the world. Overall, these findings suggest however, ELA disrupts this process, diminishing the perceived value of adaptive exploration and risk-taking, impeding a flexible view of the world, and restricting behavioral responses to challenges (Fig. 3a).

While no rodent studies have specifically examined the impact of ELA on the development of mPFC-NAc circuits, some research has explored how stress influences this circuit’s role in behavior. Social isolation for 8 weeks during early life (P21–P77) leads to a reduction in social recognition, even following 4 weeks of resocialization. Reduced social recognition in this group was associated with reduced excitability of cells in the infralimbic subregion of the mPFC that project to the NAc, and activation of this pathway rescues deficits in behavior [177]. Neurons in the mPFC that project to the NAc have been shown to guide an approach to a reward cue [57, 178]. Chronic stress reduces the activity of this cell population to the reward cue and leads to a reduction in approach behavior [179]. These studies highlight the role of mPFC-NAc driving motivated behavior and the potential for stressors to blunt activity in these pathways, leading to deficits in reward processing, social interactions, and goal-directed behavior.

## Conclusions and future research directions

While behavioral changes in adolescence likely promote learning that affords independence, adolescence is also when maladaptive behaviors tend to arise in individuals afflicted with mental health disorders. Cognitive behavioral therapy, a common treatment for anxiety- and stress-related disorders, is less effective in young individuals compared to in adults [180, 181]. Optimizing therapies for treating the developing brain requires a deeper understanding of the developmental circuit mechanisms that drive threat and reward behaviors. The preclinical, cross-species behavioral studies we reviewed here suggest that the brain systems that promote emotional learning, decision-making, reward-seeking, and threat avoidance function differently in different developmental stages. Their extended development and late sensitive windows make frontolimbic circuits exceptionally vulnerable to early disruptions such as altered caregiving. While we are only beginning to understand mPFC sensitive periods, it is becoming increasingly clear that different circuit elements are likely plastic in distinct developmental windows.

Achieving a deeper understanding of the nature of these periods, including which circuit elements are developing at different times, and how the coordinated development of different circuit elements influences behavior, is key for developing new interventions. For instance, while it is now clear that the activity-dependent development of mPFC PV+ cells and MD-mPFC circuits have profound impacts on cognitive flexibility in adulthood [43, 44], the developmental trajectories within those circuits remain poorly understood. Which types of synaptic connections form in different developmental windows? How do cell-intrinsic properties and encoding of behavioral variables change across development (Fig. 3c)? Answering these kinds of questions will allow a deeper understanding of how activity during sensitive windows influences cognition and behavior.

Here we focused on frontolimbic circuits as they mediate behavioral functions that are often disrupted in stress-related disorders. It is becoming increasingly clear that mPFC, NAc, BLA, and the connections between them develop at different rates. Therefore, for disease risk factors like ELA, stressors of different nature, timing, and duration will produce different outcomes for neural circuit development and related behaviors. To develop better therapies, it is critical that preclinical studies consider the development of mPFC, key target regions, and their connections at once, and draw parallels between humans and rodent processes whenever possible. Viral-genetic approaches for circuit mapping have been increasingly applied to the developing rodent brain, enabling researchers to finely dissect development processes with high spatial and temporal precision. It is now clear that there is incredible heterogeneity within projection-defined populations of mPFC neurons. For instance, neurons that project to NAc contain multiple anatomical classes with collateral projections to different subsets of target regions [66]. Future studies that characterize developmental timelines in frontolimbic circuits can focus on this heterogeneity. Manipulating and recording activity in distinct classes of developing mPFC projection neurons during behavior will be key for revealing the cellular and circuit mechanisms through which risk factors like ELA can alter developmental trajectories, leading to mental health disorders (Fig. 3c).
